# Heart failure with preserved ejection fraction beyond the heart: exploring the heart–liver–pancreas axis

**DOI:** 10.1093/eschf/xvag040

**Published:** 2026-01-30

**Authors:** Han Naung Tun, Omar Rahal, Ivan R Figueroa Baez, Omar Santana-Sánchez, Timothy Gardner

**Affiliations:** Geisel School of Medicine at Dartmouth, Hanover, NH, USA; Bouve College of Health Sciences, Northeastern University, Boston, MA, USA; English Division, Medical University of Warsaw, Warsaw, Poland; San Juan City Hospital Internal Medicine Training Program, San Juan, Puerto Rico; VA Caribbean Healthcare System, Internal Medicine Training Program, San Juan, Puerto Rico; Center for Digestive Health, Dartmouth-Hitchcock Medical Center, Lebanon, NH, USA

**Keywords:** Heart failure with preserved ejection fraction (HFpEF), Hepato–pancreato–cardiac axis, Systemic inflammation, Metabolic dysfunction-associated steatotic liver disease (MASLD), Pancreatic exocrine insufficiency

## Abstract

It has become increasingly recognized that heart failure with a preserved ejection fraction (HFpEF) results from an inflammatory process, congestion, and metabolic dysbiosis rather than an intrinsic structural heart abnormality. Recent studies have highlighted the close link between the heart, the liver, and the pancreas, which are organically connected via the same inflammatory processes and pathways. Liver congestion and fibrosis are responsible for the inflammatory process and the lack of metabolic adaptation, whereas pancreatic ischaemia and insufficiency of the exocrine glands aggravate malnutrition and cachexia, worsening the heart condition. Acute pancreatitis may cause heart failure and arrhythmia through injury and systemic inflammatory response syndrome (SIRS), an inflammatory process mediated by the cytokines released during the injury process. Recognition of this hepato–pancreato–cardiac axis offers a paradigm shift towards integrated management of HFpEF, emphasizing anti-inflammatory, metabolic, and haemodynamic interventions. Future research integrating multi-organ imaging, inflammatory biomarkers, and therapeutic trials such as GLP-1 receptor agonists, SGLT2 inhibitors, and cytokine blockers will be critical to disrupt this tri-organ inflammatory circuit and improve outcomes in HFpEF.

## Graphical Abstract

**Graphical Abstract:**
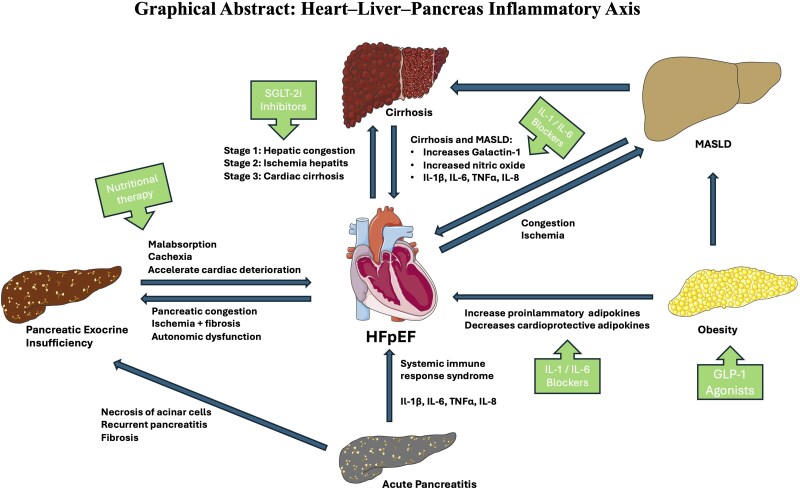
Heart–liver–pancreas inflammatory axis. Image(s) provided by Servier Medical Art (https://smart.servier.com), licenced under CC BY 4.0 (https://creativecommons.org/licenses/by/4.0/).

## Final manuscript

Heart failure with preserved ejection fraction (HFpEF) is being increasingly recognized as a disease with inflammatory and metabolic components. This narrative review will discuss complex bidirectional interactions including hepatic-cardiac axis, pancreatic-cardiac axis, and hepatic-pancreatic-cardiac axis in patients with heart failure.

## Hepatic manifestations of heart failure

In HFpEF, impaired ventricular relaxation and reduced myocardial compliance results in chronic elevations in left atrial pressure, which is transmitted retrograde into the pulmonary circulation. Persistent rise in pulmonary venous pressure results in post-capillary pulmonary hypertension and progressive right ventricular (RV) dysfunction.^1^ As RV function declines, central venous pressure increases causing hepatic venous congestion and sinusoidal dilatation. The liver, lacking venous outlet valves, is particularly susceptible to this pressure overload, leading to hepatocellular hypoxia and centrilobular necrosis. Over time, chronic congestion promotes fibrosis and the development of so-called cardiac cirrhosis. These haemodynamic alterations exemplify how even with preserved systolic function, diastolic stiffness in HFpEF can drive significant hepatic injury.

**Figure 1:**
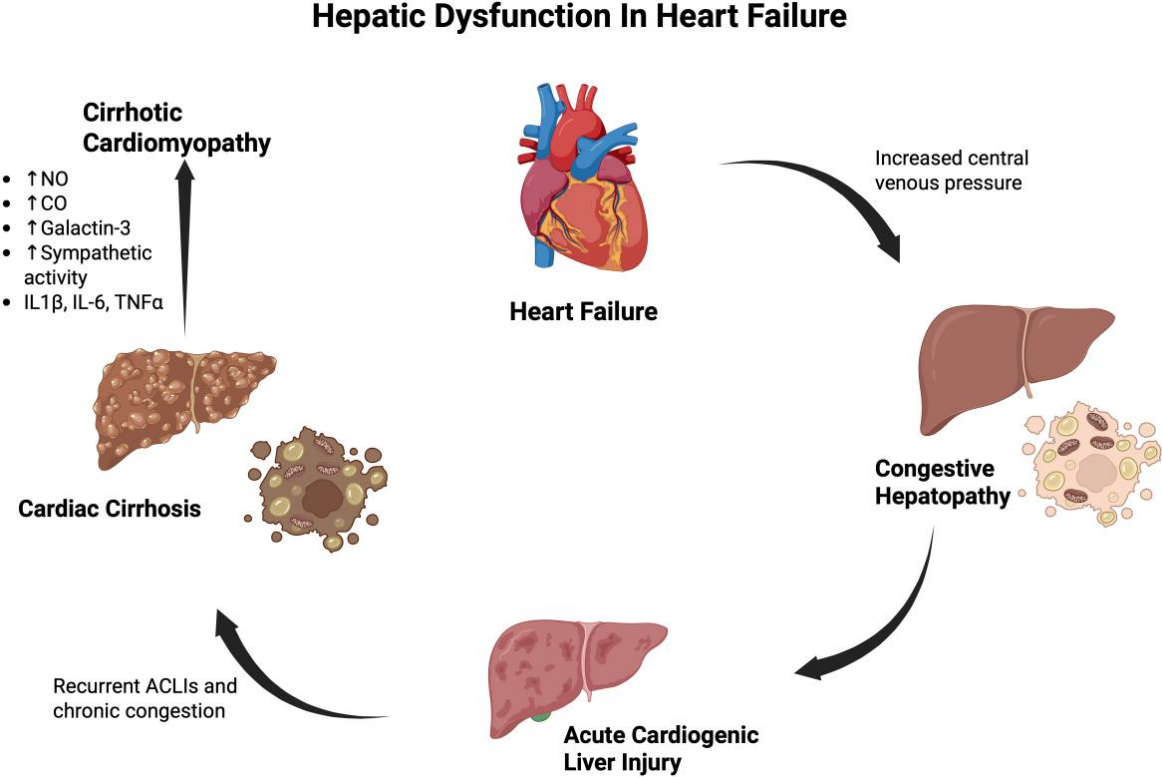
Proposed model illustrating heart–liver axis in patients with heart failure including congestive hepatopathy, acute cardiogenic liver injury, and cardiac cirrhosis. Resultant cardiac cirrhosis can exacerbate heart failure causing cirrhotic cardiomyopathy. Created in BioRender. Rahal, O. (2025) https://BioRender.com/u3cv6z8

The liver has a complex vasculature, and it receives about 25% of the cardiac output. It receives blood from the hepatic artery and portal vein and empties blood into the inferior vena cava via the hepatic veins. The hepatic artery also has a buffer system which allows it to compensate for changes in the blood flow of the portal vein.^1^ Heart failure is associated with three main hepatic complications: congestive hepatopathy, acute cardiogenic liver injury (ACLI), and cardiac cirrhosis,^2^ as shown in *Figure 1*. Hepatic veins have no valves, making them susceptible to any increase in venous pressure. In heart failure, elevated right atrial pressure and central venous pressure leads to backflow of blood from the superior vena cava to the valveless hepatic veins leading to hepatic venous congestion. This results in hepatic sinusoidal dilation and hepatic hypoxia.^1,2^ The centrilobular hepatocytes or hepatocytes in zone 3 are most affected as they are closest to hepatic venules. Zone 3 is also the primary site for detoxification, and ischaemia could potentially be a site for oxidation.^1,2^ This damage can extend to other zones and eventually the deposited collagen, connective tissue and fibrosis bridges one central vein to another leading to cardiac cirrhosis.^3^ Furthermore, chronic hepatic congestion damages the bile duct by endothelial damage and loss of tight junctions between hepatocytes leading to cholestasis.^4^ Cholestasis can then lead to bile duct obstruction and eventually lead to biliary pancreatitis, a common cause of acute pancreatitis (AP).^5^

Heart failure can also cause ACLI, commonly referred to as ischaemic hepatitis, hypoxic hepatitis, or shock liver. Acute cardiogenic liver injury occurs due to acute decompensated heart failure or cardiogenic shock. Traditionally, it was believed that cariogenic shock was caused by severely low cardiac output or sudden hypotension leading to reduced hepatic perfusion and ischaemia, especially in Zone 3. New evidence suggests that ACLI pathogenesis is contingent on the coexistence of hepatic congestion or portal hypertension.^1^ In addition, individuals with chronic congestive hepatopathy or portal hypertension are at higher risk of developing ACLI, and they may experience ACLI after mild haemodynamic disturbances. After restoration of blood to the liver, reperfusion injury can occur secondary to ACLI.^1^ Long standing heart failure can lead to cardiac cirrhosis, the third liver manifestation caused by heart failure. This is the result of chronic congestion and recurrent ACLI.^1^

Approximately 50% of patients with liver cirrhosis develop cirrhotic cardiomyopathy (CCM).^1^ Previously, CCM was characterized by both systolic and diastolic dysfunction as well as prolonged QT intervals.^1^ New evidence supports that CCM is a form of HFpEF characterized by diastolic dysfunction and/or slight systolic dysfunction.^6^ This has been attributed to the pro-inflammatory state of cirrhosis that can result in cardiomyocyte apoptosis and isoform switching of myosin heavy chain from the A subtype to the weaker B subtype.^1,7^ Furthermore, cirrhotic liver releases toxins which reduce systemic vascular resistance leading to a hyperdynamic pulse, which can then progress to a degree where the heart is unable to maintain effective circulatory volume.^1,7^

Multiple contributors have been implicated in the development of CCM including elevations in nitric oxide, carbon monoxide, and galactin-3. For instance, cirrhotic liver produces excess amounts of nitric oxide which causes widespread reduction in systemic vascular resistance.^7–9^ In addition, tumour necrotic factor alpha along with other inflammatory cytokines induce the expression of Nitric Oxide Synthase 2, boosting nitric oxide production.^7^ Endogenous carbon monoxide production is also increased in cirrhosis due to sympathetic activation, and it is associated with reduction of ventricular contractility.^7^ Galactin-3 is a biomarker of inflammation and fibrosis of cardiac tissue. Galactin-3 levels are found to be high in patients with cirrhosis.^7,10^ Another study found that galactin-3 inhibits cardiac contractility via TNF-a, and that inhibition of galactin-3 reduced cardiac TNF-a and brain natriuretic peptide, and it improved in systolic function and blood pressure.^10^

## Integrated heart–pancreas–inflammation axis in acute pancreatitis and heart failure with preserved ejection fraction

Acute Pancreatic Events & Cardiac Consequences (with emphasis on HFpEF)

Acute pancreatitis is a complex disease with both local biliary-pancreatic as well as systemic effects. Localized effects on pancreatic and biliary parenchyma have been well described, both on a short-term scale, such as complications including peripancreatic fluid collections and necrosis, as well as on a long-term scale, including chronic pancreatitis, fibrosis, and pancreatic exocrine and endocrine insufficiency.^11^ Systemic effects are seen on virtually every organ system; however, most of the described pathologies remain acute or sub-acute in their presentation.^12^ Examples range from systemic inflammatory response syndrome (SIRS), acute respiratory distress syndrome, shock, arrhythmias, electrocardiological changes, disseminated intravascular coagulation, and metabolic derangements, to more uncommon manifestations, such as pancreatic encephalopathy and rhabdomyolysis, among others.^12^ Several inflammatory pathways have been described that could explain both localized and systemic effects in the setting of AP. Even more, anti-inflammatory therapy has been studied for the prevention of damage in such systems, with even the proposed use of natural compounds as medical therapy.^13^ However, the extent of such systemic inflammation sequelae in the long term has been characterized to a lesser degree.

**Figure 2:**
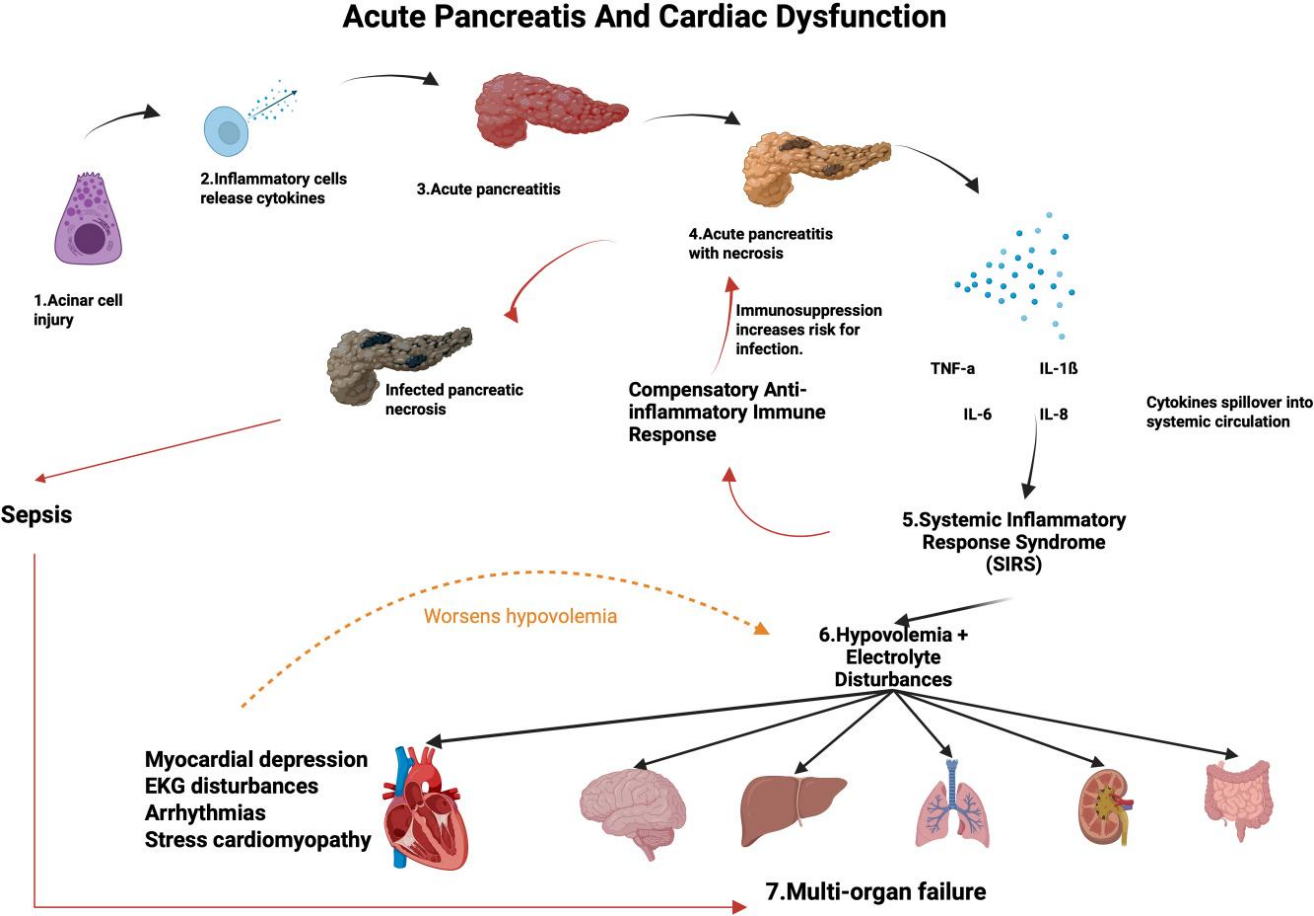
Diagram illustrating the pathogenesis of acute pancreatitis and how spill-over of inflammatory cytokines causes SIRS resulting cardiovascular manifestations. After a strong immune response such as SIRS, a compensatory anti-inflammatory response increases the risk of developing infected pancreatic necrosis which can potentially lead to sepsis and worsening of multi-organ disturbances. Created in BioRender. Rahal, O. (2025) https://BioRender.com/c6zymrb

The leading causes of AP are alcohol consumption and gallstones,^14^ and other rarer causes including hypertriglyceridemia, post-retrograde cholangiopancreatography and drug induced AP.^5^ The pathophysiology of AP is divided into three stages: (i) intrapancreatic trypsinogen activation; (ii) intrapancreatic inflammation, and the presence of varying degrees of pancreatic necrosis; and (iii) progression of AP, and development of SIRS.^12^ The proposed mechanism of AP and heart failure is shown in *Figure 2*. Initial acinar injury caused by intrapancreatic trypsinogen activation activates series of other zymogens such as elastases and phospholipases which damage pancreatic parenchyma and peripancreatic fats releasing damage-associated molecular patterns (DAMPs).^12,15^ These DAMPs then induce a local inflammatory response where macrophages release high levels of pro-inflammatory cytokines including interleukin (IL)-1β, tumour necrosis factor (TNF)-α, IL-6, and IL-8.^12,15^ Interleukin-6 is of particular importance as it is closely associated with multi-organ involvement and complications of AP.^5^ As this local inflammation progresses, spill-over of cytokines in systemic circulation leads to the development of SIRS, fever, tachycardia, increased vascular permeability, vasodilation leading to hypovolemia and hypertension. Severe SIRS can lead to early multi-system failure including cardiovascular, respiratory, renal, and hepatic failure.^5^ Other evidence reveals that AP can cause cardiac manifestations at any stage of AP.^12^ For instance, electrolyte disturbances can arise by nausea and vomiting from AP, and cardiac depression may arise from cardio-biliary reflex.^12,16^

An important patient population with high vulnerability for such sequelae involves those with HFpEF. Moreover, this population also includes those with baseline risk factors for developing HFpEF, such as older age, hypertension, dyslipidaemia, diabetes, and obesity.^17^ It was previously believed that myocardial depressant factor (MDF) and the vascular entry of pancreatic enzymes were the main cause of cardiac dysfunction in AP.^14^ Specifically, MDF was believed to directly reduce cardiac contractility while pancreatic enzymes damage cardiac vasculature leading to a reduction in cardiac perfusion and coronary vasospasm.^14^ Physiologically, the endothelium produces endothelium-derived substances which can cause vasodilation, anti-proliferative, and anti-thrombogenic as well as substances that mediate vasoconstriction, prothrombotic, and proliferative potential. Damage of the endothelium can lead to imbalanced secretion of these substances and is associated with coronary vasospasm.^18^ As previously stated, current medical literature supports that myocardial depression is primarily driven by SIRS,^12,14^ a systemic inflammatory response characterized by the release of DAMPs, proteases, and cytokines such as IL-1, IL-6, and TNF-α.^19^ Pro-inflammatory cytokines, including IL-1B and TNF-a, act directly on ventricular myocytes and decrease myocardial contractility.^20,21^ This inflammatory cascade exerts significant strain on the cardiovascular system through various mechanisms. The inflammatory surge promotes a mix of distributive shock due to systemic vasodilation and cardiogenic shock by decreasing myocardial contractility. Catecholamine excess and electrolyte disturbance, such as hyperglycaemia and hypocalcaemia, can induce cardiac arrhythmias and further decompensate heart function.^19^ The same inflammatory cytokines have been proposed to cause endothelial activation and microvascular dysfunction, leading to myocardial stiffness, a deleterious mechanism in HFpEF, where diastolic reserve is already limited. Accelerated atherosclerosis further contributes to long-term effects on cardiovascular disease and heart function.^19^

Cytokine storms in SIRS also increases vascular permeability and systemic vasodilation, which leads to ‘third spacing phenomenon’, shifting plasma into interstitial spaces, leading to hypotension and tachycardia.^5,22^ This leads to a systemic hypoperfusion which can exacerbate multi-organ ischaemia. Hypovolemia-induced hypoxia can lead to ischaemia, increase reactive oxygen species, and eventually lead to a secondary activation of inflammatory pathways amplifying SIRS. This forms a bidirectional loop worsening the already-existing hypovolemia and SIRS.^12^ For instance, kidney hypoxia can result in metabolic disturbances including hypocalcaemia, hypomagnesemia, and hypophosphataemia adding to the metabolic disturbances in heart failure patients.

Manifestations of cardiac disease in AP include cardiovascular depression, metabolic disturbances which lead to new or worsening of arrhythmias, and changes in vasomotor tones of peripheral vessels. The most common EKG findings include T-wave changes and depression of the ST-segment, and they occur in 50% of the patients.^12^ In patients with pre-existing cardiac abnormalities, AP behaves as a stressor which aggravates and potentially triggers symptoms of imminent cardiovascular diseases.^5^ Case reports of Takotsubo-like cardiomyopathy during episodes of AP further highlight the vulnerability of the cardiovascular system, even in patients without risk factors or previous heart disease.^12,23^ However, it is believed that these pancreatitis-induced cardiac manifestations can be reversed after treating AP.^5,12^ Cohort studies have shown that cardiovascular abnormalities are associated with worse outcomes in patients with AP, and the severity of AP is directly correlated with the severity of cardiovascular failure.^14^ A large national study in Germany from the year 2005–19 which included 797 364 hospitalization-cases due to AP. From the cohort, a total of 50 749 had heart failure, 44 925 survived and 5824 died during their hospitalization, and scientists deduced that heart failure had an odds ratio of 2.3 of in-hospital mortality^5^ illustrating the severity and complex interactions between AP and heart failure. SIRS remains the primary driver of AP-induced cardiac dysfunctions. Some studies have shown that after a very strong inflammatory response such as SIRS, the body responds with a compensatory anti-inflammatory response via the secretions of IL-10 and TGF-B. This can weaken the immune system and increase the risk of infection of the damaged pancreas leading to sepsis, a subtype of SIRS,^24–26^ as shown in *Figure 2*. Sepsis can then lead to or even worsen the multi-organ failure.

Obesity and visceral/epicardial adipose tissue, either by themselves or under the umbrella of metabolic dysfunction-associated steatotic liver disease (MASLD), release pro-inflammatory adipokines, linking metabolic dysfunction to diastolic impairment.^27,28^ With MASLD already being an independent risk factor for increasing cardiovascular risk aside from traditional risk factors and being strongly linked to HFpEF development, it is thought to act systemically as an amplifier of inflammation, oxidative stress, and fibrosis that can worsen both cardiac and pancreatic function.^29^ Subsequently, patients with HFpEF exhibit chronic low-grade inflammation, endothelial dysfunction, and microvascular changes that leave them vulnerable to an exacerbated risk of suffering myocardial stiffness, pulmonary congestion, and arrhythmias during episodes of AP. Catecholamines and inflammatory-mediated stress in HFpEF patients may present with disproportionate haemodynamic instability, causing a decrease in stroke volume, EKG changes, and impaired diastolic dysfunction.^12^ Studies found that in patients with AP, up to 67% had increased pro-brain natriuretic peptide levels, and 48% had abnormal echocardiographic findings, with diastolic dysfunction present in up to 83% either isolated or in combination with systolic dysfunction,^30,31^ further accentuating the vulnerability of HFpEF patients in this scenario.

Obesity and visceral adiposity are believed to lead to altered synthesis of adipokines. A new publication by Milton Packer divides adipokines into three primary domains. Domain 1 adipokines are produced in healthy non-obese individuals, and they include adiponectin, C1q related proteins such as CTRP3 and CTRP9, extracellular nicotamide phosphoribosyl transferase, Secreted Frizzled-related protein 5, zinc alpha-2 glycoprotein, and neuregulin-4.^32^ Domain 1 adipokines are secreted by healthy adipocytes, and they are cardioprotective as they alleviate stress and reduce systemic inflammation. Domain II adipokines cardioprotective, and they are upregulated in obese patients, and they encompass fibroblast-growth factor-21, metallothionein, irisin, hepatocyte growth factors, and adiponeurotrophins.^32^ Their function is to counteract the hypertrophic, metabolic, and inflammatory stress in both adipose tissue and the heart.^32^ Domain 3 adipokines are upregulated in obese individuals and are secreted by hypertrophic and inflamed adipocytes, and they are pro-inflammatory, pro-hypertrophic, pro-fibrotic, and anti-natriuretic adipokines properties.^32^ These include but are not limited to leptin, resistin, and asprosin. Obesity is associated with a reduction in domain adipokines and elevation of domain III adipokines.^32^ In essence, domain II adipokines compensate for the reduction of domain I adipokines and compensate for the elevation of domain III adipokines, but this compensation is inadequate and insufficient to stop the progression of HFpEF.^32^ Bariatric surgery has been associated with restoration of domain I and II adipokines, and reduction of domain III adipokines.^32^ Heart failure with preserved ejection fraction is highly prevalent in women in comparison to men. This can be elucidated by the fact that women have higher levels of adipose tissue which can directly correlate with higher levels of domain III adipokines.^32^

## Impact of heart failure with preserved ejection fraction on pancreatic function

**Figure 3:**
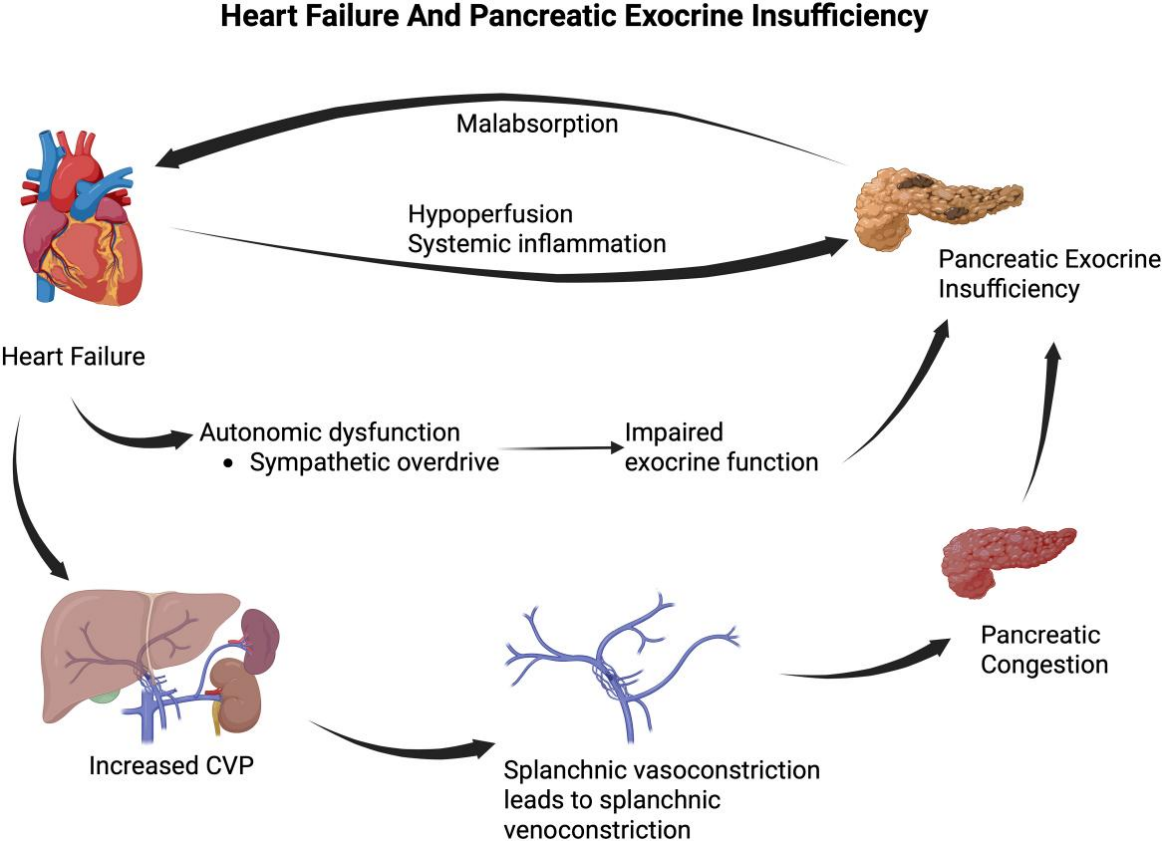
Proposed model of the heart–liver–pancreatic exocrine insufficiency axis in heart failure. Heart failure causes systemic inflammation, autonomic dysfunction, pancreatic hypoperfusion, and congestion all of which contribute to the pathogenesis of pancreatic exocrine insufficiency. Pancreatic exocrine insufficiency causes malabsorption of micronutrients worsening the cachexia and metabolic decline which may worsen heart failure. Created in BioRender. Rahal, O. (2025) https://BioRender.com/07qhwc9

The pancreas is a complex multifunctional organ with endocrine and exocrine functions. The Islet of Langerhans, which is dispersed within exocrine tissue, represents only 5% of the pancreatic volume. These islets are responsible for the secretion of various hormones including insulin and glucagon. The exocrine component of the pancreas accounts for 95% of the pancreatic volume. They are composed of acinar and ductal cells which produce pancreatic enzymes and bicarbonates which eventually get deposited in the proximal duodenum.^15,33^ Research suggests that the most common pancreatic manifestation in heart failure is pancreatic exocrine insufficiency (PEI), and rarely AP. Heart failure leads to systemic inflammation, reduction in pancreatic perfusion, venous congestion of organs including the pancreas, and autonomic dysfunction^34^ which all combined lead to PEI.^33^ Ageing as well is a risk factor, as ageing also leads to acinar cells atrophy.^33^ The complex interplay between heart failure and PEI is shown in *Figure 3*.

The pancreas has a very complex and relatively ischaemic-resistant blood supply. Its arterial supply forms a circuit formed by the anastomosis between the coeliac and the mesenteric arteries. Blood flow to the pancreas is about 1% of the cardiac output in healthy adults, with the venous drainage being entirely to the portal system through the splenic and superior mesenteric veins.^33^ A decreased cardiac output leads to a decrease in perfusion of the pancreas resulting in a pancreatic ischaemic injury. In addition, increased intraventricular pressure in heart failure results in increased central venous pressure preventing drainage of abnormal organs including splanchnic venous congestion. Elevations in venous pressure are maintained solely by increased oxygen extraction, and due to a lack of compensatory autoregulatory mechanisms, when portal pressures increase beyond specific thresholds, pancreatic blood flow is reduced.^33^ In settings of expanded intravascular volume, such as in patients with congestive heart failure (CHF), the compliance of the splanchnic veins is decreased due to splanchnic venoconstriction which is an attempt to mobilize blood to the heart. Splanchnic venoconstriction combined with elevated central venous pressue, further reduces venous drainage of abdominal organs and worsening existing pancreatic congestion.^33^ Additionally, pronounced vasoconstriction occurs due to strong adrenergic response, organs in the abdominal compartment including the pancreas, become more prone to congestion.^33^ Interestingly, although the major effect of systemic vasoconstriction is usually mediated by the sympathetic system, the disproportion in response from the mesenteric circulation is thought to be due to the renin-angiotensin axis because of higher affinity to Angiotensin II by the mesenteric vascular smooth muscle.^33^ This provides an additional outlet that could be exploited when developing therapeutic options.

Literature on pancreatic effects on the heart during acute insults is present, although further characterization is still needed. However, when it comes to heart effects upon the pancreas in patients with heart failure, especially HFpEF, the literature is sparse. Various mechanisms have been hypothesized to contribute to this interaction. Studies have found that in patients hospitalized with CHF, PEI was found in about 57% of them. Research suggests that there is a paroxysmal loss of pancreatic exocrine function with each episode of acute heart failure. Therefore, in the presence of chronic heart failure, there is a chronic functional decline in the exocrine function of the pancreas leading to long-term exocrine pancreatic insufficiency. In addition, the low cardiac output state in heart failure is associated with increased sympathetic activity and decreased parasympathetic activity which can also impair exocrine secretion in the pancreas.^34–36^ Pancreatic exocrine insufficiency is defined by decreased faecal elastase-1 levels, and an inverse relation was found between the increasing severity of CHF and the levels of faecal elastase-1.^19,37,38^ Likewise, HFpEF frequently involves venous congestion, elevated central pressures that extend to the portal and splanchnic circulation, autonomic dysfunction, and reduced perfusion, which leads to pancreatic ischaemia and exocrine dysfunction.^19^

**Figure 4:**
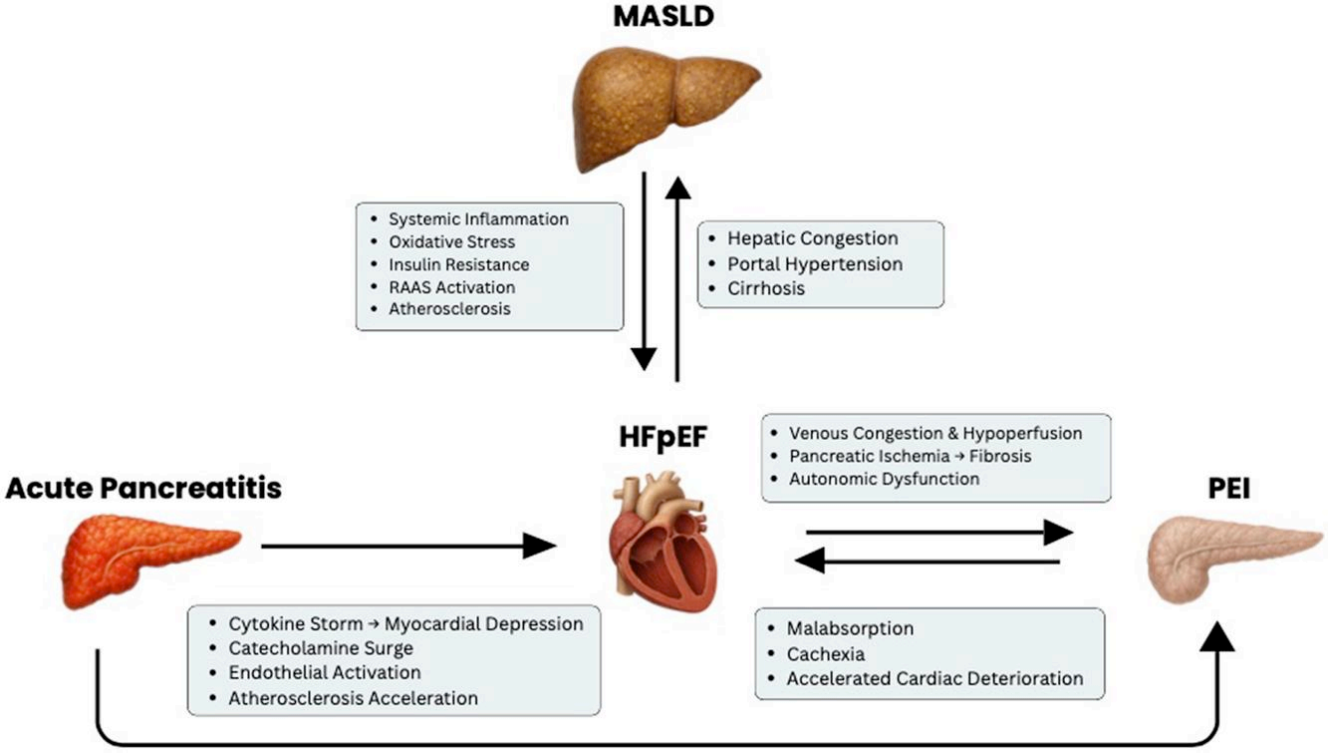
Proposed integrated heart–liver–pancreas inflammation axis. Acute pancreatitis has a unidirectional inflammatory relationship with heart failure with preserved ejection fraction and pancreatic exocrine insufficiency, while the latter two form a vicious loop with continuing deconditioning of both organs. Metabolic dysfunction-associated steatotic liver disease underlying metabolic interaction further contributes to the development of heart failure with preserved ejection fraction and indirectly exerts deleterious effects on the pancreas

An extra layer of complexity is added when considering long-term AP sequelae. Pancreatic exocrine insufficiency can occur in up to 35% of all patients with AP,^11^ with some patients developing new-onset diabetes or PEI after AP. HFpEF and pancreatic interactions highlight that reduced splanchnic flow and congestion may impair digestive enzyme secretion and nutrient absorption, contributing to cachexia and metabolic decline. In this scenario, PEI can aggravate the wasting of advanced HF and may result in micronutrient deficiencies, further deteriorating heart failure.^19^ This loop is emphasized in *Figure 4*, where different unidirectional and bidirectional interactions are proposed as major contributors to the heart–pancreas inflammation axis. Pancreatic dysfunction in heart failure has been poorly described in the literature, probably due to the complexity of measuring the functionality and perfusion of human pancreatic tissue.^8^ Therefore, more research is needed to accurately characterize this phenomenon and develop strategies to mitigate disease progression.

## Therapeutic implications (targeting both organs and the inflammatory ‘third axis’)

Specific data regarding treatment or prevention of the hepato–pancreatic–heart inflammation axis remains to be developed. Nonetheless, knowledge regarding individualized systems exists and is being studied on a larger scale. Lifestyle modifications and metabolic interventions have been proposed to be the cornerstone in preventing long-term inflammatory sequelae in MASLD and cardiovascular disease. Glucagon-like peptide-1 (GLP-1) receptor antagonists have been demonstrated to be effective in achieving weight loss, improving cardiorespiratory fitness, and lowering systemic inflammation in obese patients with HFpEF phenotype, while SGLT-2 inhibitors have reduced Heart failure hospitalizations, along with providing benefits in renal and metabolic systems.^27,28^

Targeted therapy against cytokine blockade (e.g. IL-1, 1L-6) can be helpful in preventing AP cytokine storm effects on the cardiovascular system, as well as chronic MASLD systemic inflammatory effects,^39,40^ emerging as a possible key component of HFpEF care in the future. Pancreatic enzyme replacement and targeted protein diets could improve nutrient absorption and prevent the downward spiral of cachexia and metabolic effects as part of the HFpEF-PEI loop. However, extensive clinical studies are still needed. On a final note, redirecting RAAS therapy to the Pancreas as a primary focus, either during episodes of AP or in patients with venous congestion due to heart failure, could help develop an additional outlet for treatment and prevention. Proposed therapeutics are shown in *Table 1*.

**Table 1:** Proposed therapeutic nodes and mechanisms of action as options to be studied in the future for further characterization

Therapeutic node	Mechanism
**GLP-1 receptor agonists (GLP-1 RA)**	Decreases adiposity; decreases inflammation
**SGLT2 inhibitors (SGLT2i)**	Decreases congestion; reduces heart failure hospitalizations; preserves β-cell function
**IL-1/IL-6 blockers**	Reduces inflammatory signalling
**Pancreatic Enzyme Replacement Therapy/nutritional therapy**	Corrects pancreatic exocrine insufficiency (PEI) and cachexia
**Renin-Angiotensin-Aldosterone System antagonists**	Alleviate disproportionate vasoconstriction of mesenteric vascular smooth muscle

## Future research directions

Future investigations should deepen understanding of systemic inflammation as the central mediator linking cardiac, hepatic, and pancreatic dysfunction in the HFpEF spectrum. Prospective studies are needed to evaluate pancreatic enzymes (amylase, lipase, faecal elastase-1) together with inflammatory mediators such as CRP, IL-6, and TNF-α as predictors of HFpEF outcomes following AP.^12,19,27^ Advanced imaging modalities—particularly magnetic resonance imaging-proton density fat fraction and elastography—should explore correlations between hepatic and pancreatic steatosis, parenchymal fibrosis, and myocardial stiffness to better characterize the systemic metabolic phenotype.^27,29,41^ Mechanistic intervention trials assessing anti-inflammatory and metabolic therapies, including GLP-1 receptor agonists and SGLT2 inhibitors, in patients with overlapping MASLD, HFpEF, and pancreatic insufficiency are critical to elucidate potential synergistic benefits across organs.^27^ Developing integrated multidisciplinary care models combining cardiology, hepatology, gastroenterology, and endocrinology is essential to holistically manage this tri-organ axis and mitigate inflammation at its origin.^29,42^ Because pancreatic enzyme insufficiency and hepatic congestion often coexist yet remain underdiagnosed, future research should prioritize standardized diagnostic protocols combining clinical, biochemical, and nutritional assessment with functional testing.^33^ Likewise, imaging techniques such as endoscopic retrograde cholangiopancreatography, computed tomography, endoscopic ultrasound, and magnetic resonance imaging can detect structural abnormalities—ductal changes, hyperechoic regions, cysts, and calcifications—that help predict exocrine insufficiency and define the chronic inflammatory burden shared by the pancreas and liver.^33^

## Conclusion

The interconnectedness of the *heart–liver–pancreas axis* underscores the importance of viewing HFpEF not as an isolated cardiac disorder but as a systemic inflammatory and metabolic syndrome. The coexistence of MASLD, pancreatic dysfunction, and diastolic heart failure creates a tri-organ network in which oxidative stress, endothelial injury, and cytokine activation perpetuate reciprocal organ damage. Hepatic steatosis contributes to systemic inflammation and metabolic inflexibility, the pancreas amplifies this dysfunction through impaired endocrine and exocrine activity, and the heart, already burdened by diastolic stiffness, suffers from heightened haemodynamic stress and microvascular inflammation. Recognizing this integrated pathophysiology invites a paradigm shift towards early identification and simultaneous targeting of all three organs through anti-inflammatory, metabolic, and haemodynamic therapies. In addition, obesity is associated with alteration in adipokines secretion where domain I cardioprotective adipokines are suppressed and pro-inflammatory domain III adipokines synthesis is heightened, and the compensatory release of cardioprotective domain II adipokines is unable to counteract the elevation of pro-inflammatory adipokines. Ultimately, advancing research into this multi-organ inflammatory circuit will pave the way for precision medicine strategies capable of interrupting the congestion, fibrosis, and inflammation cycle—thereby improving prognosis and quality of life for patients with HFpEF and its hepatic–pancreatic counterparts.

## Declarations

### Data Availability

No data were generated or analysed for this manuscript.
